# Single‐Cell *XIST* Expression in Human Preimplantation Embryos and Newly Reprogrammed Female Induced Pluripotent Stem Cells

**DOI:** 10.1002/stem.1992

**Published:** 2015-05-21

**Authors:** Sharon F. Briggs, Antonia A. Dominguez, Shawn L. Chavez, Renee A. Reijo Pera

**Affiliations:** ^1^ Department of GeneticsInstitute for Stem Cell Biology and Regenerative Medicine, Stanford UniversityStanfordCaliforniaUSA; ^2^ Department of Obstetrics and GynecologyInstitute for Stem Cell Biology and Regenerative Medicine, Stanford UniversityStanfordCaliforniaUSA

**Keywords:** X chromosome inactivation, Preimplantation embryo development, *XIST* RNA, Human, Human induced pluripotent stem cells

## Abstract

The process of X chromosome inactivation (XCI) during reprogramming to produce human induced pluripotent stem cells (iPSCs), as well as during the extensive programming that occurs in human preimplantation development, is not well‐understood. Indeed, studies of XCI during reprogramming to iPSCs report cells with two active X chromosomes and/or cells with one inactive X chromosome. Here, we examine expression of the long noncoding RNA, *XIST*, in single cells of human embryos through the oocyte‐to‐embryo transition and in new mRNA reprogrammed iPSCs. We show that *XIST* is first expressed beginning at the 4‐cell stage, coincident with the onset of embryonic genome activation in an asynchronous manner. Additionally, we report that mRNA reprogramming produces iPSCs that initially express *XIST* transcript; however, expression is rapidly lost with culture. Loss of *XIST* and H3K27me3 enrichment at the inactive X chromosome at late passage results in X chromosome expression changes. Our data may contribute to applications in disease modeling and potential translational applications of female stem cells. Stem Cells *2015;33:1771–1781*

## Introduction

One X chromosome in female placental mammals is transcriptionally inactivated in order to equalize gene expression between sexes 1. In the mouse, the paternal X chromosome is inactivated in the preimplantation embryo and developing extra‐embryonic tissues 2; during blastocyst formation, the paternal X chromosome is reactivated within the inner cell mass (ICM) 3, 4. Random X chromosome inactivation (XCI) is then initiated in the developing epiblast and is stably inherited in all daughter cells 5. The long noncoding RNA, *Xist*, controls XCI in *cis* by mediating gene silencing on the inactive X chromosome 6, 7, 8, 9. In contrast to XCI in the mouse, much less is known of human XCI. *XIST* RNA has been detected in whole human embryos as early as the 1‐ to 8‐cell stages using polymerase chain reaction (PCR) analysis and/or fluorescence in situ hybridization (FISH) 6, 10, 11, 12, 13. However, it remains unclear whether all cells of the human embryo express *XIST* or if expression varies between blastomeres upon *XIST* expression initiation.

Similarly, the status of *XIST* expression in human embryonic stem cells (hESC) and human induced pluripotent stem cells (iPSCs) is not clear and is reported to be highly variable 14, 15. In the mouse ESC, derived from the ICM, are *Xist* negative and maintain two active X chromosome 16. Likewise, mouse iPSCs derived from somatic cells that express *Xist* reactivate their inactive X chromosome upon reprogramming 17. However, several groups have demonstrated lack of X chromosome reactivation in humans, with continued *XIST* expression from fibroblasts to iPSCs 18, 19, 20, 21, 22. In contrast, others have documented loss of *XIST* and reactivation of the silent X chromosome that can be transient 23 or stably propagated 24, 25, 26, 27. Established hESC lines also display variable *XIST* expression as a function of extended culture and/or early derivation conditions 28, 29. As loss of XIST expression may be correlated with increases in oncogene expression 30, it remains important to understand *XIST* expression dynamics in these therapeutically relevant cells.

Here, we characterized *XIST* expression in single cells through the first days of preimplantation human embryo development and at early and late time points following mRNA reprogramming of female fibroblasts, a reprogramming method likely to be preferred due to the absence of genomic integration of reprogramming factors. We use single cell quantitative real time PCR (qRT‐PCR) to characterize *XIST* expression throughout early embryogenesis and provide a comparison of preimplantation human development with single, newly reprogrammed female iPSCs. We demonstrate that single blastomeres of the 4‐cell embryo begin to express *XIST*, and continued expression of *XIST* is asynchronous. We also show that single mRNA reprogrammed iPSCs express *XIST* at early passage (P0), and that the percentage of single cells expressing *XIST* decreases over time in culture. The cells that lose *XIST* expression undergo a loss of H3K27me3 enrichment at the inactive X chromosome in addition to X‐linked gene expression changes.

## Materials and Methods

### Sample Source

Human embryos were obtained from two sources and have been described in detail 31, 32, 33. All embryos were from successful *in vitro* fertilization (IVF) cycles and donated for non‐stem cell research, with informed consent from the Stanford University RENEW Biobank. Deidentification and molecular analysis were performed according to the Stanford Institutional Review board (IRB)‐approved protocol #10466 entitled “The RENEW Biobank” and the University of Minnesota IRB‐approved protocol #0306M49242 entitled “Stage‐Specific Genomic Characterization of Human Preimplantation Embryos.” No protected health information was associated with each of the embryos.

### Human Embryo Culture

Human embryos were cultured as described previously 30, 31. Thawed embryos were placed in a polystyrene dish containing 0.5 M sucrose solution for 10 minutes, then 0.2 M sucrose solution for a subsequent 10 minutes. Next, embryos were washed with Quinn's Advantage Medium with HEPES (Cooper Surgical, Trumbull, CT, http://www.coopersurgical.com) with the addition of 5% Quinn's Advantage Serum Protein Substitute (Cooper Surgical). Embryos were cultured in either Quinn's Advantage Cleavage or Blastocyst Medium (depending on stage) plus 10% Serum Protein Substitute (Cooper Surgical) under mineral oil (Sigma, St Louis, MO, http://www.sigmaaldrich.com) at 37°C with 6% CO_2_, 5% O_2_, and 89% N_2_ under standard human embryo culture conditions and in agreement with current clinical IVF practice. The zona pellucida was removed by treatment with Acidified Tyrode's Solution (Millipore, Billerica, MA, http://www.millipore.com), and single blastomeres were collected by incubating in Quinn's Advantage Ca2+ and Mg2+‐free medium with HEPES (Cooper Surgical) for 5–20 minutes at 37°C with pipetting to break up into single cells. Blastomeres were tubed and flash frozen at −80°C until qRT‐PCR analysis.

### Cell Culture

The adult female primary fibroblast line (30 year old female) was isolated and cultured as previously described 33. Fetal fibroblasts were obtained from ATCC (WS1, CRL‐1502). Fibroblasts were cultured on 0.1% gelatin‐coated dishes in mouse embryonic fibroblast (MEF) media. Media was changed every 1–3 days, and cells were passaged with TripleE (Life Technologies, Rockville, MD, http://www.lifetech.com) when confluence reached 80%–100%. iPSCs were cultured in feeder‐free conditions on Matrigel (BD Biosciences, San Diego, CA, http://www.bdbiosciences.com) coated plates and passaged manually with glass tools.

### mRNA Reprogramming

The protocol used was adapted from Stemgent's mRNA reprogramming protocol (http://assets.stemgent.com/files/1359/original/ProtocolMicroRNA_GK_061314.pdf). Approximately 5.0 to 5.7 × 104 fibroblasts were plated in one well of a six‐well dish coated with Matrigel (BD Biosciences) in MEF media (10% FBS, 1% Pen/Strep in DMEM [Dulbecco's modified Eagle's medium] + GlutaMAX) (Gibco, Grand Island, NY, http://www.invitrogen.com). After 24 hours, media was changed to nuFF‐conditioned pluriton media supplemented with B18R (eBioscience, San Diego, CA, http://www.ebioscience.com) and Pluriton supplement (Stemgent, San Diego, CA, https://www.stemgent.com). Cells were equilibrated in 6% CO_2_, 5% O_2_, and 89% N_2_ for 2 hours, then transfected with mRNA for *OCT4, SOX2, KLF4, c‐MYC,* and *LIN28* using Stemfect transfection reagent (Stemgent). Transfections continued daily for 11 days with nuFF‐conditioned pluriton media changes prior to transfection. On days 1 and 5, miRNAs (Stemgent) were added to the mRNA cocktail for transfection.

### NuFF Conditioned Media

Briefly, 4 million human NuFF cells were plated in a T75 flask in MEF media (10% fetal bovine serum (FBS), 1% Pen/Strep in DMEM + GlutaMAX) (Gibco). After 24 hours, media was removed and 25 ml of pluriton media (Stemgent) was added. Every 24 hours following the initial change, 25 ml of condition media was collected, filtered, and stored and replaced with 25 ml fresh media until 150 ml had been collected.

### *XIST* RNA FISH

iPSCs were passaged onto Matrigel‐ (BD 354230) coated two to four well chambered slides; fibroblasts were passaged onto gelatin‐coated slides. Cells were cultured for 1–4 days until cells had attached and grown to suitable size/confluence. Media was removed, and cells were washed once with phosphate buffered saline (PBS). A 1 ml fixation solution (4% paraformaldehyde electron microscopy sciences [EMS], Hatfield PA, http://www.emsdiasum.com/microscopy/ in PBS [Gibco]) was added to the cells for 10 minutes at room temperature. Fixation solution was removed, and cells were washed twice with PBS. Cells were then permeabilized with 70% ethanol for 1 hour to overnight at 4°C. After removing 70% ethanol, 1 ml wash buffer: 10% formamide (Fisher Scientific, Hampton, NH, http://www.fisherscientific.com), 1× saline‐sodium citrate bugger (VWR, Radnor, PA, https://us.vwr.com) brought up in nuclease free water was added to the cells and incubated for 2–5 minutes. Wash buffer was aspirated, and 10–20 µl of probe (Biosearch Technologies, Petaluma, CA, https://www.biosearchtech.com) diluted 1:50 for a final concentration of 500 nM in hybridization buffer: dextran sulfate (1 g) (Millipore), 1× SSC (VWR), 10% formamide (Fisher Scientific), brought up in nuclease free water was added to the slide. To prevent drying, a coverslip was added, and cells and incubated in a dark humidified chamber overnight at 37°C. The next day, the coverslips were removed, and cells were incubated in wash buffer for 30 minutes at 37°C. DAPI was added to the wash buffer and incubated for 5 minutes before prolong gold (Life Technologies) was added to the slides and mounted with coverslips. Slides were dried for 1–4 hours in the dark at room temperature prior to imaging. Slides were imaged on a Zeiss LSM 700 confocal microscope, and images were processed using ImageJ.

### Fluorescence Activated Cell Sorting Analysis for SSEA4 and TRA‐1–60

Colonies were dissociated to single cells by treatment with Accutase (Fisher) for 3–5 minutes. Cells were washed with 10 ml mTeSR (StemCell Technologies, Vancouver, BC, Canada, http://www.stemcell.com) media, filtered on a 70 mm strainer (BD Bioscience), spun down for 5 minutes at 1,000 rpm, and resuspended in FACS buffer (1× PBS, 0.1% bovine serum albumin, EDTA) to a concentration of 1 × 106 cells per 100 µl. Cells were blocked with 0.8% mouse IgG (Invitrogen) then incubated with TRA‐1–60 (BD Biosciences) and SSEA4 (BD Biosciences) or TRA‐1–60 live stain (Stemgent) antibodies for 30 minutes on ice and protected from light. The cells were then washed with fluorescence‐activated cell sorting (FACS) buffer and resuspended in FACS buffer + DAPI. Flow‐cytometry analysis was performed on a BD FACS AriaII cell sorter. Compensation beads (BD Biosciences) were used to ensure proper staining patterns during data acquisition. Dead cells and doublets were gated out, and PE‐TRA‐1–60 versus FITC‐SSEA4 was then used to identify a double positive and double negative (fibroblast) population, which was then purity sorted before cells were single cell sorted into individual wells of 12‐8 strip PCR tubes with 5 µl of CellsDirect 2× reaction mix solution (Invitrogen, Carlsbad, CA, http://www.invitrogen.com).

### qRT‐PCR Analysis

Embryos were either analyzed using gene‐specific Taqman Probes (Applied Biosystems, Foster City, CA, http://www.appliedbiosystems.com) as described in previous work 30 or gene‐specific primers and EvaGreen DNA‐binding dye 34 (Biotium, Hayward, CA, http://biotium.com). All primer pairs were designed to span exons and initially tested using genomic DNA samples and showed either a *C*_t_ value > 26 or had no amplification (data not shown). Human iPSCs were analyzed with EvaGreen primers. Single cells were preamplified for specific targets by addition of SuperscriptIII RT Platinum Taq Mix (Invitrogen) and 200 nM mixture of pooled primers. Cells underwent an Exonuclease I (New England BioLabs, Ipswich, MA, https://www.neb.com) reaction to remove unincorporated primers. STA‐ExoI samples were diluted 1:2 with DNA suspension buffer (Teknova, Hollister, CA, http://www.teknova.com), and qRT‐PCR was performed in the presence of EvaGreen DNA binding dye with the Biomark HD system and 96.96 Dynamic Arrays (Fluidigm, South San Francisco, CA, https://www.fluidigm.com).

### Single Cell Data Processing

Single cell qRT‐PCR data were initially filtered by determining the correct melting temperature for each gene, exporting *C*t data into Excel (Microsoft) as Heat Map Results and removing data with a *C*t call that failed. The secondary filter consisted of removing *C*t values > 26, which was determined as the limit of detection (LOD) for this set of assays (data not shown). Cells with low or no expression of housekeeping genes were removed for single fibroblast and iPSC studies but were included in single blastomere analysis. Log2Ex values were then determined by LOD (26)—*C*t Log2Ex values were then normalized by cell size by taking the difference in the mean expression for single cells and the average mean of each cell on the chip (Normalized Log2Ex values).

### Statistical Analysis of X Chromosome Expression

Single cell expression values were split into *XIST*+ and *XIST*− populations and graphed on a density plot by gene. To compare the distribution of the values of a single gene, both populations were adjusted to equal areas under the curve. The means of both populations were graphed and, using a Welch's two‐sample *t* test, the differences in the means were computed.

## Results

### *XIST* Expression in Whole Human Preimplantation Embryos and Individual Blastomeres

**Figure 1:**
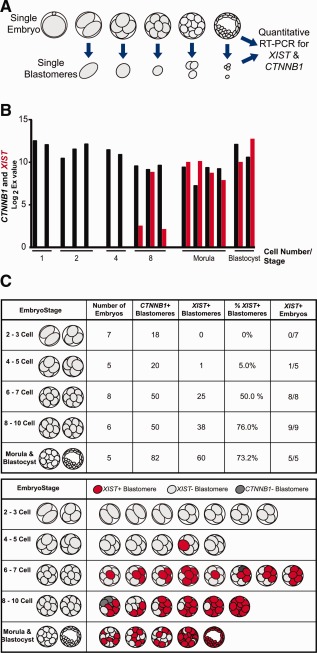
*XIST* expression in whole embryos and single cells during human preimplantation development. **(A):** Schematic of *XIST* and housekeeping gene, *CTNNB1*, qRT‐PCR on whole and single or grouped blastomeres of preimplantation human embryos. **(B):** Log2Ex values for *XIST* (red) and *CTNNB1* (black) from single preimplantation human embryos from the 1‐cell to blastocyst stage. **(C):** Table of *XIST* and *CTNNB1* expression in single or grouped blastomeres from five stages of preimplantation development (2–3 cell, 4–5 cell, 6–7 cell, 8–10 cell, and morula–blastocyst stages). Schematic demonstrates *XIST+* blastomeres (red), *XIST−* blastomeres (light grey), and nonviable blastomeres, *CTNNB1−* (dark grey). Abbreviation: qRT‐PCR, quantitative real‐time polymerase chain reaction.

We first examined expression of *XIST* during human preimplantation embryo development in whole embryos from the 1‐cell to blastocyst stages via qRT‐PCR (Fig. 1A). Expression of the housekeeping gene, *CTNNB1*, measured as Log2Ex values (fold change above background), was used to evaluate embryo viability as previously described 30. Whole embryos between the 1‐ and 4‐cell stages had expression of *CTNNB1* but did not have detectable *XIST* transcript (Fig. 1B). The major wave of embryonic genome activation (EGA) occurs on day 3 (4–8‐cell stage) of embryo development 35, and at the 8‐cell stage, embryos exhibited both *CTNNB1* and *XIST* expression. Expression of *XIST* was also found in embryos at the morula and blastocyst stages of development (Fig. 1B).

Recent studies suggest that individual human blastomeres have asynchronous gene expression during development 30, 34. Therefore, we examined *XIST* expression in single blastomeres of a new set of embryos during the first days of human development to determine if *XIST* is similarly expressed in an asynchronous manner (Fig. 1A). Single blastomeres isolated from 2‐ to 3‐cell embryos demonstrated high expression of *CTNNB1*, but no detectable *XIST* expression, consistent with the whole embryo data (Fig. 1C; Supporting Information Fig. S1A). At the 4‐ to 5‐cell stage, one blastomere from a single 4‐cell stage embryo, out of five embryos analyzed, had detectable *XIST* transcript. From the 6‐ to 7‐cell stage, all embryos analyzed had the presence of at least one blastomere with *XIST* expression, with 50% of *CTNNB1+* blastomeres expressing *XIST*. However, the percentage of *XIST+* blastomeres varied between embryos. By the 8‐ to 10‐cell stage, all six embryos had blastomeres with detectable *XIST* transcript, with 76.0% of *CTNNB1*+ blastomeres expressing *XIST* transcript. At the morula to blastocyst stages, all embryos had cells with *XIST* transcript, but the percentage of *CTNNB1+* cells expressing *XIST* was maintained at 73.2% (*CTNNB1*+/*XIST*+ blastomeres). Because of the method of embryo dissociation, we were unable to determine the location of *XIST*+ and *XIST*− blastomeres within the morula, but all cells (or clusters of cells) in the single blastocyst stage embryo analyzed expressed *XIST* transcript (Fig. 1C; Supporting Information Fig. S1A). Taken together, we demonstrate that *XIST* expression in individual blastomeres is not synchronized and initiation of *XIST* transcription happens in single blastomeres as early as the 4‐cell stage. A few of the embryos were also assayed for Y chromosome‐specific gene expression (*SRY, RBMY,* and *ZFY)*, but we only detected expression within one 6‐cell embryo, which expressed *XIST* within half of the blastomeres (Supporting Information Fig. S1). Statistically, we would expect half of the embryos to be male, however, it has been previously demonstrated that male embryos do express *XIST* transcript in the preimplantation stage 11, and therefore, irrespective of embryo sex. The characterization of *XIST* in a mixture of female and male preimplantation embryos is informative for X chromosome dynamics in development. However, we did not focus on determination of the sex of the embryos at these early stages.

### *XIST* Is Expressed in Newly Reprogrammed iPSCs But Lost with Passage

**Figure 2:**
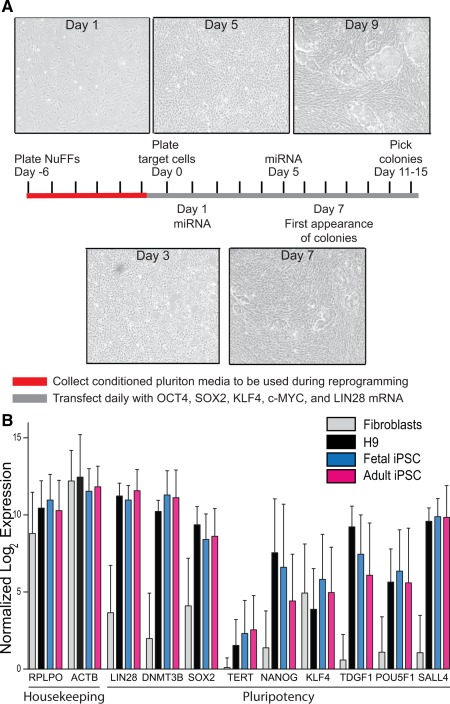
mRNA reprogramming produces fully reprogrammed colonies as early as 11 days post‐reprogramming initiation. **(A):** mRNA reprogramming protocol used to reprogram fetal and adult fibroblasts into iPSCs. The reprogramming process was only successful when supplemented with miRNAs on days 1 and 5. Images show progression of cell morphology and the appearance of colonies. Magnification 5×. **(B):** Normalized single cell expression values for housekeeping and pluripotency markers compared to an ESC control, showing that both iPSC lines are indeed pluripotent. Abbreviation: iPSC, induced pluripotent stem cell.

Multiple studies have addressed XCI in human female iPSCs following reprogramming, albeit with conflicting conclusions: either indicative of reactivation 23, 24, 25, 27, 36 or maintenance 18, 19, 20, 21, 22 of the inactive X chromosome in iPSCs. However, much of this work has focused on cells reprogrammed via methods involving viral integration of the reprogramming factors and after the cells have been in culture for a number of passages. We therefore sought to analyze *XIST* expression in single cells immediately following reprogramming of female fibroblasts and used the nonintegrating mRNA reprogramming method. To this end, we derived iPSCs from two female fibroblast lines one fetal (WS1, CRL‐1502) and one adult (30 year old female; Fig. 2A). Our protocol derived iPSCs without feeders or reprogramming factor integration, eliminating the effects of these variables on *XIST* expression in newly reprogrammed iPSCs. In addition, all reprogramming occurred under physiological (5%) O2 and required the addition of Stemgent miRNAs on two days of the reprogramming process (Day 1 and 5). We observed that colonies of reprogrammed cells arose as early as day 7 following the first transfection of mRNA for *OCT4, SOX2, KLF4, c‐MYC,* and *LIN28* and were large enough to be manually picked between days 11 and 15 (Fig. 2A). iPSCs derived by this method express similar levels of multiple pluripotency genes when compared to H9 hESCs (Fig. 2B; Supporting Information Fig. S2B, S2C) and maintain an ESC morphology at early and late passages (Supporting Information Fig. S2A).

**Figure 3:**
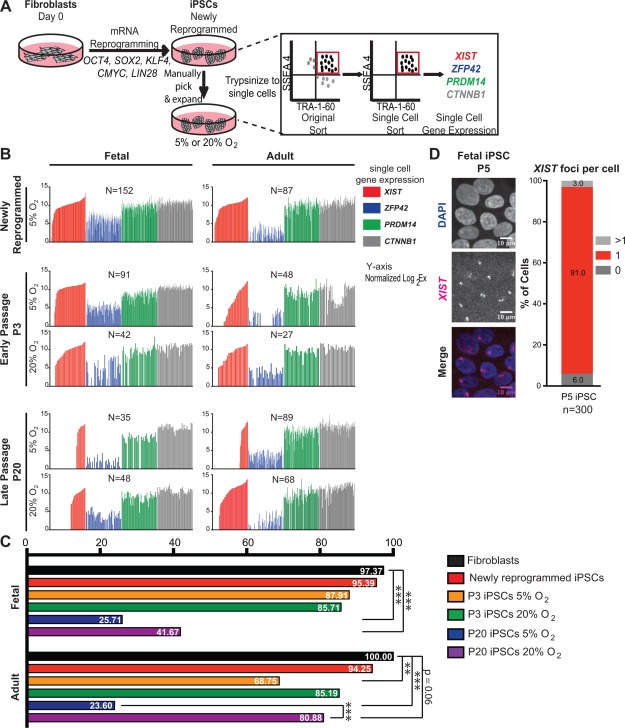
*XIST* expression in single cell newly reprogrammed female iPSCs. **(A):** Schematic for isolating pluripotent cells from a heterogeneous reprogramming well. Following successful reprogramming, some cells were manually passaged and analyzed at later passages, while the remainder were fluorescence‐activated cell‐sorted and analyzed by quantitative real‐time polymerase chain reaction for single‐cell gene expression. **(B):** Normalized log2Ex values for newly reprogrammed fetal and adult iPSCs. **(C):** Percentage of single cells expressing *XIST*. Loss of *XIST* is first significant, relative to the parental fibroblasts, at passage 20 in fetal cells and at passage 3 in adult cells (only in 5% O_2_) using Fisher's test: **, *p* < .01; ***, *p* < .001. **(D):** RNA fluorescence in situ hybridization for *XIST* in female fetal iPSCs. Abbreviation: iPSCs, induced pluripotent stem cells.

To assess the *XIST* expression in newly reprogrammed iPSCs, we sorted TRA‐1–60/SSEA4 double positive pluripotent 37 single cells at passage 0; these cells had been in culture between 11 and 15 days following the initiation of reprogramming but had not yet been passaged (Fig. 3A). We analyzed more than 1,750 single cells by qRT‐PCR to assess *XIST* expression, the housekeeping gene *CTNNB1*, and two pluripotency markers *ZFP42* (*REX1*) and *PRDM14*, to ensure that FACS isolated cells were enriched for pluripotent cells (Fig. 3A, 3B). We demonstrate that fetal and adult P0 single iPSCs expressed *XIST* (95.39% and 94.25%, respectively), similar to the percentages seen in the parental fibroblasts (fetal, 97.37% and adult, 100%), indicating that newly mRNA reprogrammed iPSCs maintain *XIST* expression (Fig. 3B, 3C). We further supported our qRT‐PCR‐based analysis of *XIST* expression with RNA fluorescence in situ hybridization (RNA FISH). We observed that iPSCs expressed *XIST* in the majority (94%) of cells and in a manner characteristic of the inactive X chromosome in both cell lines as well as at both 5% and 20% O2, consistent with our gene expression analysis (Fig. 3D; Supporting Information Fig. S3).

### Oxygen Tension Affects *XIST* Expression in Cell Lines Differently Over Time

Oxygen levels at time of derivation and during prolonged culture have been shown to cause different XCI states in human ES cells 28, 29. Thus, to determine the effect of oxygen tension on *XIST* expression in mRNA reprogrammed iPSCs, we expanded our P0 newly reprogrammed iPSCs at either physiological (5%) or atmospheric (20%) O2 levels after initial derivation (Fig. 3A). The consequences of oxygen tension differed between fetal and adult cell lines. In the fetal iPSCs, differences in oxygen levels did not affect the rate at which *XIST* expression was lost in single cells. At P3, cells cultured in both 5% and 20% O2 did not significantly differ in *XIST* expression from the parental fibroblasts, although there was a slight decrease in the percentage of cells expressing *XIST* (87.91% and 85.71%, respectively; Fig. 3B, 3C). However, by P20 the percentage of *XIST*+ cells at both 5% and 20% O2 were significantly lower than the parental fibroblasts (25.71% [*p* = 2.11 × 10−14], 41.67% [*p* = 7.44 × 10−12], respectively). Surprisingly, there was no statistical difference in *XIST* expression between 5% and 20% at either P3 or P20. Alternatively, adult iPSCs cultured in 20% O2 maintained *XIST* expression at both early (P3; 85.19%) and late passage (P20; 80.88%). While statistically insignificant, the percentage of *XIST* positive cells did decrease with passaging to suggest that they are more resistant to passage‐dependent *XIST* loss but not entirely exempt from it (Fig. 3C). In contrast to fetal iPSCs, adult iPSCs in 5% O2 lost *XIST* expression more rapidly. By P3, the percentage of *XIST*+ cells was significantly different than the parental fibroblasts (68.75%; *p* = .0018) and by P20 the expression of *XIST* in adult iPSCs was only 23.60% (*p* = 1.71 × 10−15), comparable to the expression in the fetal iPSCs at P20 (25.71%; Fig. 3C). Because of this difference in response to oxygen tension, the percentage of adult iPSCs expressing *XIST* by P20 was substantially lower in 5% O2 (23.60%) as compared to 20% O2 (80.88%, *p* = 1.91 × 10−12). A similar pattern of *XIST* loss at high passage was also true of other late passage female hESCs and iPSCs lines reprogrammed using viral reprogramming methods, albeit at passages higher than P20 (Supporting Information Fig. S4A–S4C) 38. In contrast, the majority of male fibroblasts, ESCs and iPSCs did not express *XIST* (Supporting Information Fig. S4B). Importantly, our data demonstrate that the loss of *XIST* begins within the first few passages regardless of the age of the fibroblast donor. We also note subclonal variation in *XIST* expression, similar to what has been previously reported with other reprogramming methods 24. These data suggest a differential response to long‐term culture in different oxygen levels, and while our data only examines two cell lines, this effect would be an interesting area for further experiments.

### Female Fibroblasts Maintain XIST Expression Independent of Oxygen Concentration or Passage Number

**Figure 4:**
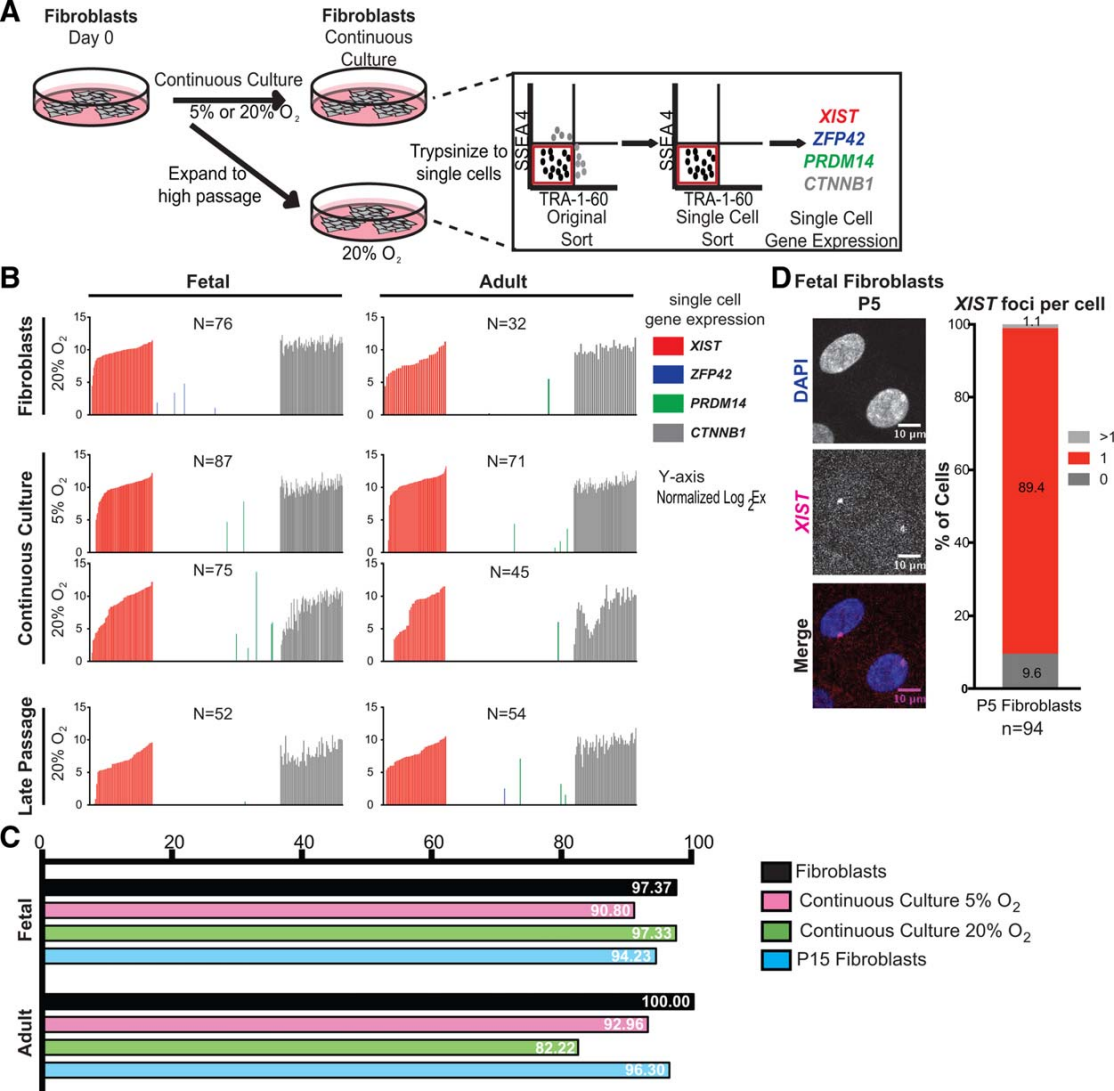
Fibroblasts maintain *XIST* expression in various passage and culture conditions. **(A):** Schematic for isolating fibroblasts after continuous culture. Some cells were passaged and analyzed at later passages. All cells were fluorescence‐activated cell‐sorted for double negative populations and analyzed using quantitative real‐time polymerase chain reaction for single‐cell gene expression. **(B):** Normalized log2Ex values for single female fibroblasts. All fibroblasts maintain XIST expression regardless of length of time in culture. **(C):** Percentage of single fibroblasts expressing *XIST*. No significant difference in percentage of *XIST* positive cells was detected in fibroblasts. Fisher test was used to determine whether groups were statistically different. **(D):** RNA fluorescence in situ hybridization for *XIST* in female fetal fibroblasts. Abbreviation: DAPI, 4′,6‐diamidino‐2‐phenylindole.

To investigate whether the loss of *XIST* expression is a result of reprogramming or culturing techniques, we cultured the parental fibroblasts under reprogramming conditions without mRNA transfections. Similar to the iPSCs, we isolated pluripotency‐negative (SSEA4−/TRA‐1–60−) fibroblasts between 11 and 15 days without passaging (Fig. 4A). Both adult and fetal fibroblasts maintained *XIST* expression during continuous culture, and that maintenance was independent of oxygen tension (Fig. 4B, 4C). Even when analyzed at late passage (P15), the fibroblasts maintained *XIST* expression and marks of an inactive X chromosome (94.23% fetal and 96.30% adult) (Fig. 4B, 4C; Supporting Information Fig. S5). Expression of *XIST* was also confirmed in the fibroblasts using RNA FISH; the vast majority of cells (89.4%) expressed *XIST* and its expression was localized to distinct foci representing the inactive X chromosome (Fig. 4D). These data suggest that the loss of *XIST* in our fetal and adult iPSC single cells is not culture or cell line dependent as the parental fibroblasts maintain *XIST* expression.

### Loss of *XIST* Expression in iPSCs Causes X Chromosome Gene Expression Changes

**Figure 5:**
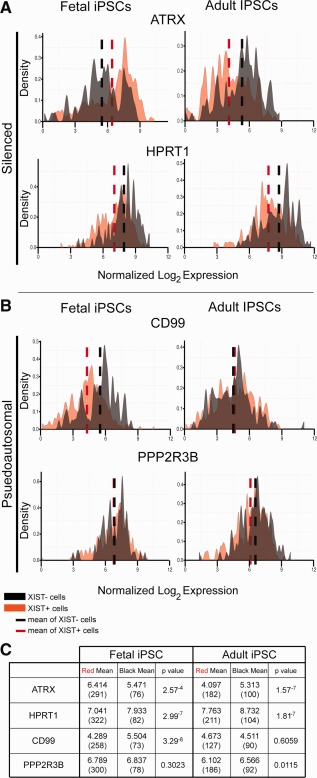
X‐linked gene expression varies with *XIST* expression. Density plot for each of four X‐linked genes from fetal and adult iPSCs were plotted with *XIST*+ populations (light red) and *XIST*‐ populations (black) separated. In adult iPSCs, there is an increase in the overall mean of silenced genes in the *XIST*− population **(A)** while the difference in means of pseudoautosomal genes is almost zero **(B)**. However, in fetal iPSCs, *ATRX* does not behave as expected (A) and there is a significant difference in one of the pseudoautosomal genes, *CD99* (B). *p* values for the differences in means was calculated using a t test (**C**). Abbreviation: iPSCs, induced pluripotent stem cells.

Because our iPSC lines rapidly lost expression of *XIST* with culture, we next sought to determine whether our single cell gene expression data could determine if the *XIST*− population in iPSCs contained a reactivated X chromosome (XaXa) or an eroded X chromosome (XaXe; cells that have lost *XIST* expression but maintain a partially silenced X chromosome with some increased X‐linked gene expression) 22. To this end, we compared the *XIST*− and *XIST*+ single cell populations and graphed a density plot of single cell expression of four X‐linked genes in both adult and fetal cell lines: *CD99* and *PPP2R3B* which escape inactivation; *HPRT1* and *ATRX* which are silenced (Fig. 5). If reactivation had taken place, the population expression mean of *XIST*− cells would be higher than the mean of *XIST*+ cells (black mean > red mean). Both silenced genes in adult iPSCs and one in the fetal iPSCs (*HPRT1*) exhibited the expected relationship (Fig. 5A). However, *ATRX* expression in the fetal iPSCs had the opposite relationship: *XIST*+ cells had higher expression compared to *XIST*− cells. When comparing the means of the two populations within a single gene, we found that the *XIST*− and *XIST*+ cells represented statistically different populations, suggesting the existence of X chromosome differences at multiple loci between *XIST−* and *XIST+* cells (Fig. 5C).

Next, we compared the expression of two genes in the pseudoautosomal regions of the X chromosome known to escape X chromosome inactivation, which should have equal expression in both *XIST*− and *XIST*+ populations. *PPP2R3B* followed the expected pattern and had almost indistinguishable expression in *XIST*− and *XIST*+ groups in both adult and fetal iPSCs (Fig. 5B). However, in adult iPSCs, *CD99* was equivalently expressed in both *XIST*− and *XIST*+ populations whereas in fetal iPSCs, the *XIST*− cells had higher expression, similar to the two silenced genes previously analyzed. Upon further comparison of the iPSCs to the parental fibroblasts, we found no significant difference in mean expression of silenced and pseudoautosomal genes in fibroblasts of either line (Supporting Information Fig. S6A–S6C). We note that the fibroblast data may be limited by the few number of cells that are *XIST*− and therefore provides a skewed representation of the mean and limited comparison value. Since the *XIST*− and *XIST*+ cell populations represent a mix of early and late passage single cells due to the fact that few cells at passage 0 are *XIST*−, observed differences may instead be due to the variations associated with passage number. Despite the statistical difference between *XIST*− and *XIST*+ cells with respect to four X‐linked genes, our single cell expression data does not definitively support the conclusion that iPSCs have reactivated their silenced X chromosome as the magnitude and direction of the changes were not consistent within or between fetal and adult iPSC lines. Rather, these data highlight the necessity to evaluate gene expression at the single cell level and suggest that certain significant epigenetic changes, consistent with erosion of XCI, occur on the X chromosome dependent on reprogramming, time in culture, and cell line.

To further probe XCI erosion, we analyzed H3K27me3, which marks heterochromatin and especially the inactive X chromosome, as well as *TSIX* expression, *XIST*'s antisense inhibitor, in late passage iPSCs. Using a Triple X female iPSC line as a control (which maintains two inactive X chromosomes in iPSCs), we showed two distinct foci of H3K27me3 staining indicative of X chromosome inactivation in these cells 38. However, neither fetal nor adult iPSCs at P20 contained H3K27me3 foci, indicating the loss of silencing marks on the inactive X chromosome (Supporting Information Fig. S7). We also analyzed *TSIX* expression, as increased levels may suggest the silencing of *XIST* on the inactive X chromosome and thus result in reactivation. We first compared mean *TSIX* expression levels across fibroblasts, P0, P3, and P20 iPSCs of combined adult and fetal lines and then separated by cell line (Supporting Information Fig. S8). P0 iPSCs had increased expression relative to the fibroblasts, and expression levels were equivalent between P0 and P20 iPSCs in both cell lines (Supporting Information Fig. S8A, S8B). However, at P3, the adult cell line had significantly decreased expression compared to P0 but not consistent with reactivation taking place (Supporting Information Fig. S8B). Altogether, our results are consistent with XCI erosion in passaged female iPSCs supported by our epigenetic and X‐linked gene expression analysis.

## Discussion

This study examines expression of the long noncoding RNA, *XIST*, in single blastomeres of the human preimplantation embryo and single female iPSCs immediately after reprogramming via mRNA and over time in culture. In single whole embryos at the 1‐ to 4‐cell stage, no detectable *XIST* transcript was observed, which is in contrast to previous studies that identified *XIST* transcript as early as the 1‐cell stage 11. *XIST* transcript was detected, however, in single blastomeres starting at the 4‐cell stage consistent with EGA and was found to be expressed in at least one or more blastomeres of embryos through the morula and blastocyst stages of development. This data is in agreement with previous studies that used *XIST* RNA FISH or nested PCR techniques 6, 12, 13. We also show that initiation and continued expression of *XIST* varies between individual blastomeres of a single embryo, which may provide insight into the X chromosome dynamics during female iPSC reprogramming.

Human cell populations are known to be heterogeneous, especially pluripotent cell types 39, 40. Single cell analysis has been used previously to characterize expression heterogeneity in cell populations 41; however, *XIST* expression in developing human embryos or newly reprogrammed female iPSCs has not been examined. Here, we use single cell analysis and observe significant cell‐to‐cell variation that would have been undetected by other methods. Single cell analysis provides insight into *XIST* expression and XCI dynamics in pluripotent stem cell derivation and embryogenesis. We also note that these methods allow assaying of multiple genes simultaneously in individual cells more readily than RNA FISH methods.

Observations regarding expression of *XIST* in single cells immediately after reprogramming extends and potentially clarifies aspects of previous studies on the X chromosome state in human iPSCs 18, 19, 20, 21, 22, 23, 24, 25, 30, 36. In particular, we find that female cells reprogrammed using an mRNA reprogramming method and analyzed at P0 are class II (*XIST*+ and maintain an inactive X chromosome) 22. Similar to other reprogramming methods 18, 22, 23 but most notably by Kim et al. 23, we show that mRNA reprogramming does not generate reprogrammed cells with two active X chromosomes, even in nascent iPSCs. However, we have not probed time points prior to colony formation, which may reveal a reactivation step that occurs during mRNA reprogramming 23. Additionally, since it has been postulated that the X chromosome state is influenced by the expression of ectopic reprogramming factors 23, 42, our approach has the benefit of avoiding the consequences of integration and the epigenetic and transcriptional abnormalities associated with ectopic expression of reprogramming factors 43. mRNA reprogramming has the useful property of easily varying the ratio and amounts of ectopic reprogramming factors and this flexibility can allow fine‐tuned control to better understand the role of reprogramming factor stoichiometry in potential X chromosome reactivation. Finally, this approach allows us to eliminate the influence of feeder cells on resulting iPSCs, providing an advantage in any future clinical translation potential of these cells 24. Therefore, it is extremely important to understand how these cells behave and evolve with time spanning initial derivation to therapy‐ready cells.

We show the loss of *XIST* expression in single, mRNA reprogrammed iPSCs as these cells are kept longer in culture. A similar phenomenon has been reported in human iPSCs reprogrammed with a number of different reprogramming techniques and is therefore not mRNA reprogramming specific 18, 22, 23. However, in addition to the dynamic changes in *XIST* expression overtime, we also profile how expression of additional X‐ linked genes change with *XIST* loss. *TSIX* expression did not change with passage, and H3K27me3 expression was lost from the inactive X chromosome. We believe this data, in conjunction with changes in expression of X‐linked genes in *XIST*− and *XIST*+ populations, supports erosion of XCI and not X chromosome reactivation. Interestingly, this raises the possibility of *XIST* expression being uncoupled from XCI at later passages when cells with *XIST* expression appear to have lost H3K27me3 expression. Our observations of a rapid loss of *XIST* expression as passage number increases 23, and, over time, further suggest that there is a departure from the expression patterns seen in human preimplantation embryos. Overall, it is clear that XCI is a complex process with significant changes occurring in *XIST*− and *XIST*+ iPSC populations that remain to be determined. Future work focusing on how to maintain or reintroduce *XIST* expression would be extremely valuable in understanding the mechanism of human XCI and for regenerative uses of female pluripotent stem cells.

## Conclusions

Data presented here using single cell analysis of *XIST* suggests that the developmental state of the human preimplantation embryo, post EGA, is marked by asynchronous *XIST* expression within single blastomeres. Additionally, nascent iPSCs are characterized by *XIST* expression that decreases over time in culture. This loss results in the erosion of X chromosome inactivation including changes in X chromosome gene expression and the loss of H3K27me3 expression from the inactive X chromosome.

## Author Contributions

S.F.B. and A.A.D.: conception and design, collection and/or assembly of data, data analysis and interpretation, and manuscript writing; S.L.C.: collection and/or assembly of data, provision of study material or patients, and manuscript editing; R.A.R.P.: conception and design, financial support, administrative support, provision of study material or patients, and final approval of manuscript. S.F.B. and A.A.D contributed equally to this work.

## Disclosure of Potential Conflicts of Interest

The authors indicate no potential conflict of interests.

## Supporting information

Supplementary Information Figures and Legends
