# Supplementary material for: Single‐Cell XIST Expression in Human Preimplantation Embryos and Newly Reprogrammed Female Induced Pluripotent Stem Cells
Source: Stem Cells. 2015 May 21;33(6):1771–81. doi: 10.1002/stem.1992 (PMC4441606; doi:10.1002/stem.1992)
Supplement: Supplementary file 1 — Supplementary Information Figures and Legends [file STEM-33-1771-s001.docx]

**Supplemental Figures and Legends**


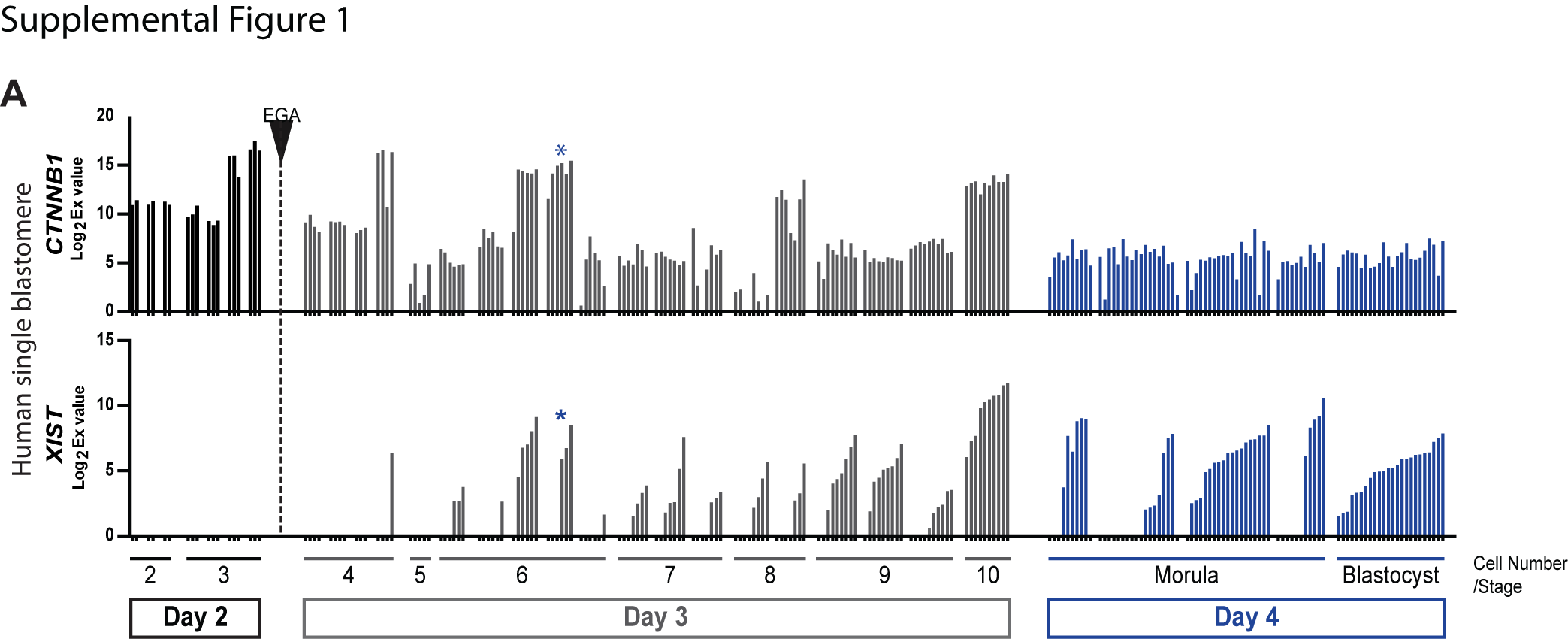


**Figure S1. *XIST* Expression in Single Blastomeres of Preimplantation Human embryos**

**(A)** Log_2_Ex Values for *CTNNB1* and *XIST* from individual blastomeres of single human embryos at day 2 (1–2-cell stage, black), day 3 (4–10-cell stage, grey) and day 4 (morula – blastocyst, blue). Blue asterisks indicate embryo had detectable *SRY* and *RBMY* transcript.


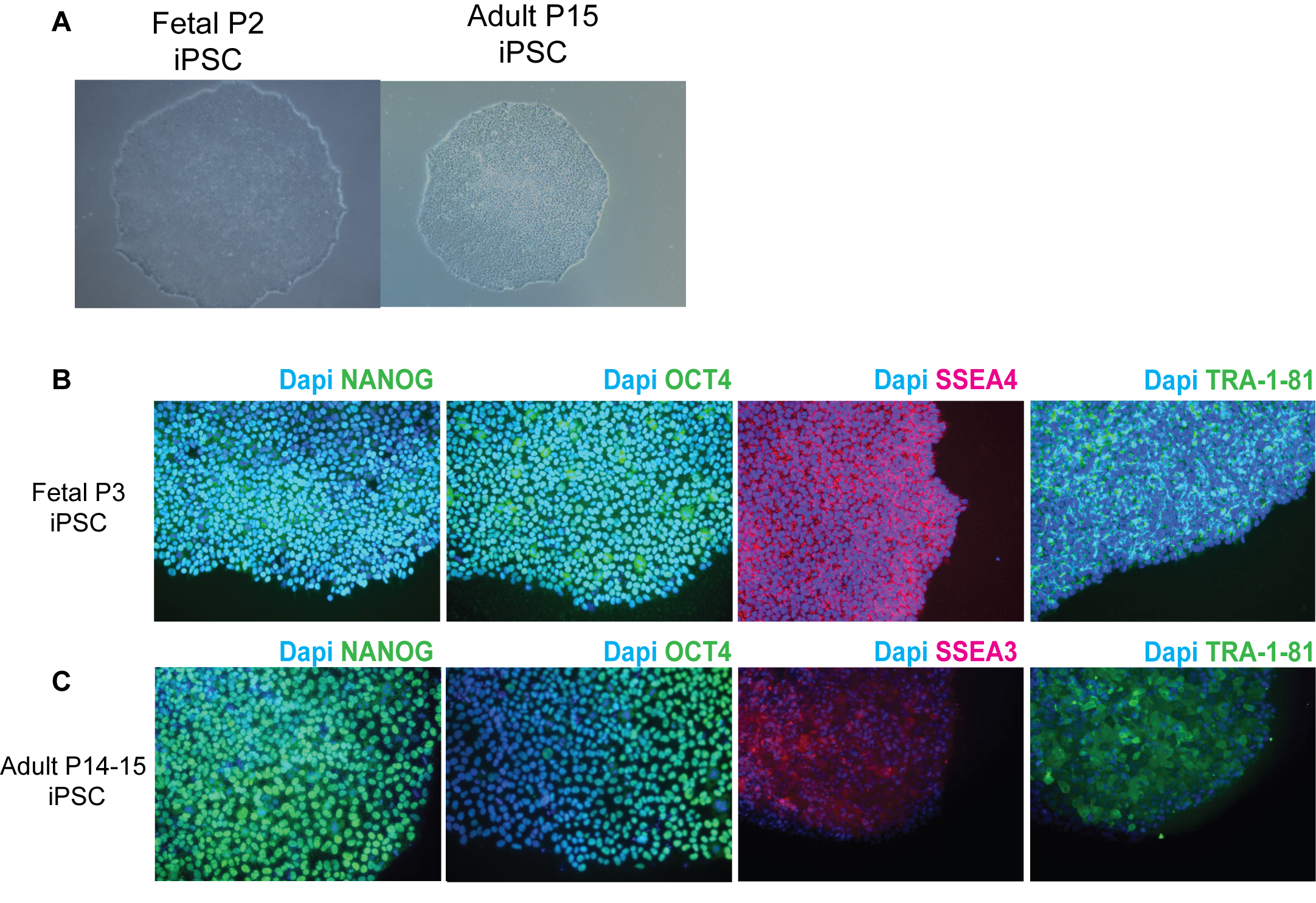


**Figure S2. mRNA reprogrammed colonies have ES cell morphology and pluripotent protein expression.**

**(A)** Early and late passage phase images of colonies derived from female fibroblasts have a characteristic iPSC morphology (5X). Note the absence of a feeder layer. Immunofluorescence images (20X) show that early and late passage colonies from both the fetal **(B)** and adult **(C)** cell lines stain positively for nuclear factors OCT4 and NANOG, as well as cell surface markers SSEA3/4 and TRA-1-81 that are characteristic of pluripotency.


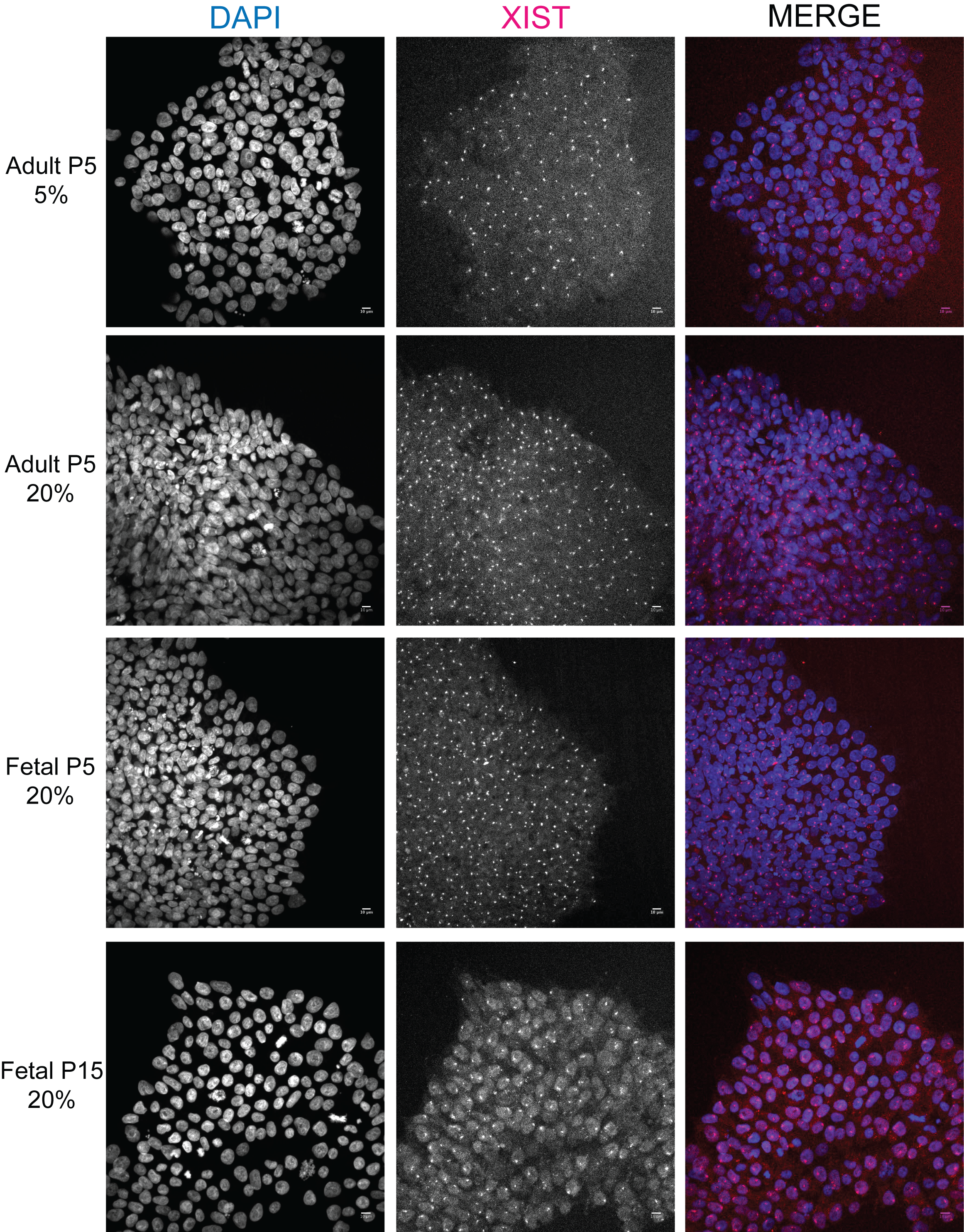


**Figure S3. RNA FISH can visualize the inactive X chromosome at various passage numbers and oxygen levels in both cell lines.**

XIST RNA FISH images of iPSC colonies showing the presence of the inactive X chromosome in both cell lines at different passages and oxygen tensions. Scale bar is 10um.


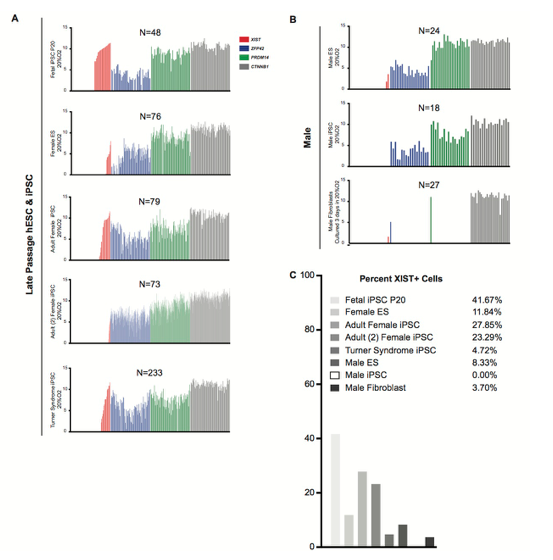


**Figure S4. Late Passage Human iPSCs and ESCs Lose *XIST* Expression.**

**(A)** Female fetal iPSCs cultured for 20 passages only express *XIST* in half the cells. This trend is more pronounced at even later passages as illustrated by the female ES and two adult female lines. All cells were cultured at 20% O_2_. **(B)** Male ES, iPSC, and fibroblasts rarely express *XIST*, as expected. **(C)** Percentage of cells expressing *XIST* for late passage female lines as well as male control lines. Longer passaging results in fewer *XIST* positive cells in female lines.


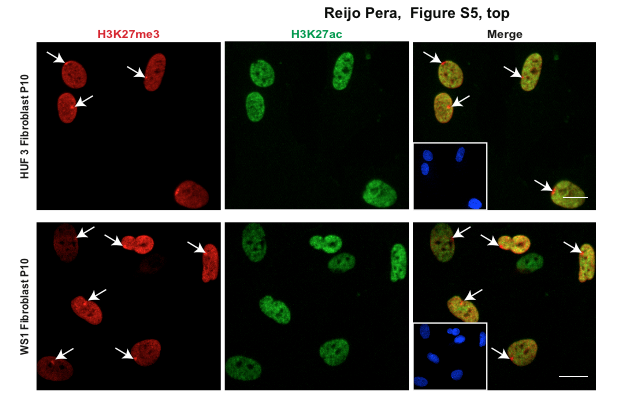


**Figure S5. HeK27me3 staining marks the inactive X chromosome**

Immunofluorescence for H3K27me3, a marker of silenced chromatin, and H3K27ac, a mark of active chromatin, clearly delineates the inactive X chromosome in female fibroblasts as a spot with high K3K27me3 expression and little to no H3K27ac. The inactive X chromosome is typically found near the periphery of the nucleus (marked by white arrows). Scale bar represents 20um.


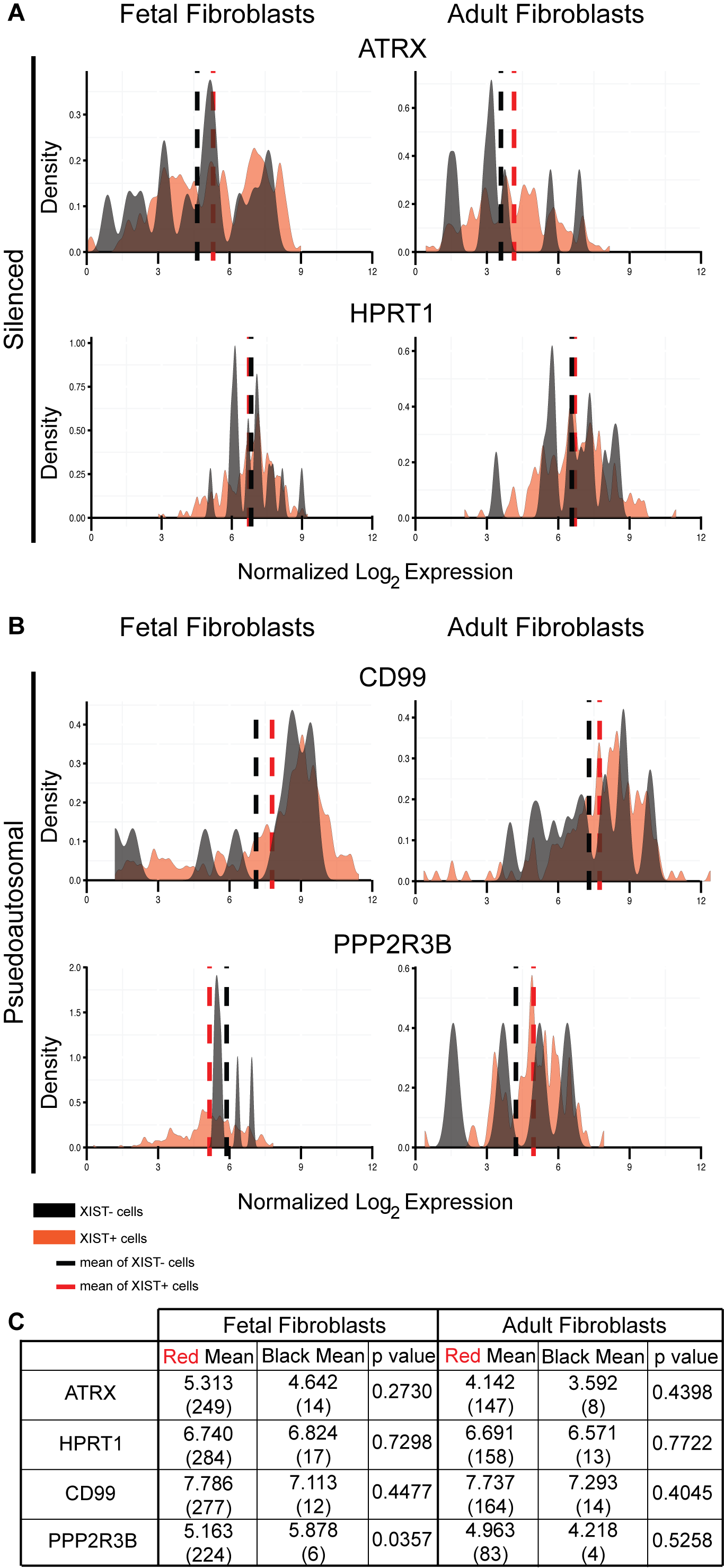


**Figure S6. X-linked genes in fibroblasts do not vary as significantly as in their iPSC counterparts.**

A density histogram for each of four X-linked genes from fetal and adult fibroblasts was plotted with XIST+ populations (light red) and XIST- populations (black) separated. Overall, the difference in the means of the XIST- and XIST+ populations is not nearly as large as for iPSCs. Neither silenced genes **(A)** nor pseudoautosomal genes **(B)** change in a direction suggesting reactivation in the XIST- group. p-values for the differences in means was calculated using a t test **(C)**.


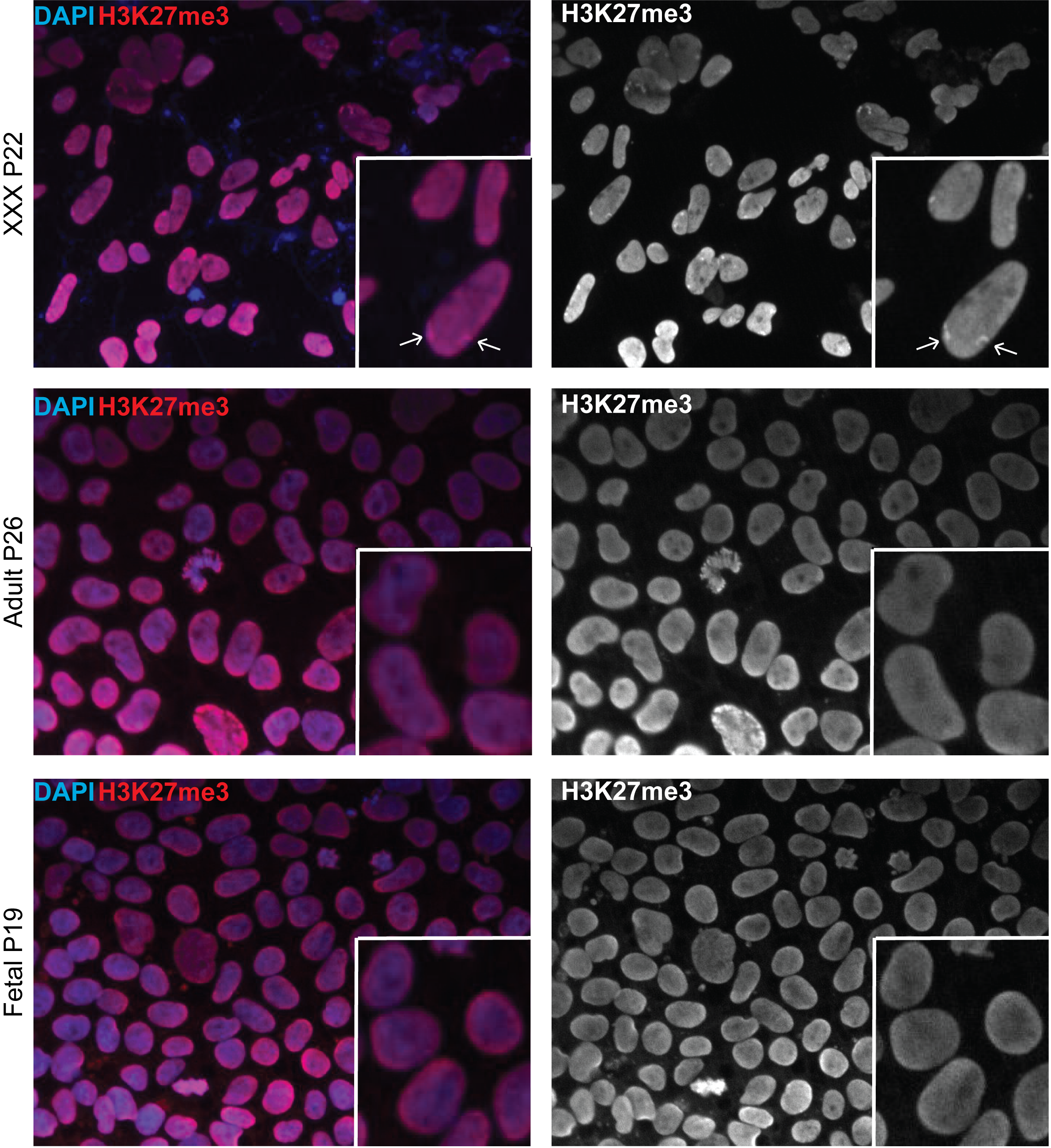


**Figure S7. H3K27me3 straining shows absence of the inactive X chromosome in late passage iPSCs.**

H3K27me3 staining, which marks the inactive X chromosome, shows two distinct foci in the XXX female control iPSC line. However, no evidence of an inactive X chromosome is seen in either the fetal or adult late passage iPSCs. All images are 40X and insets are enlarged from original images.


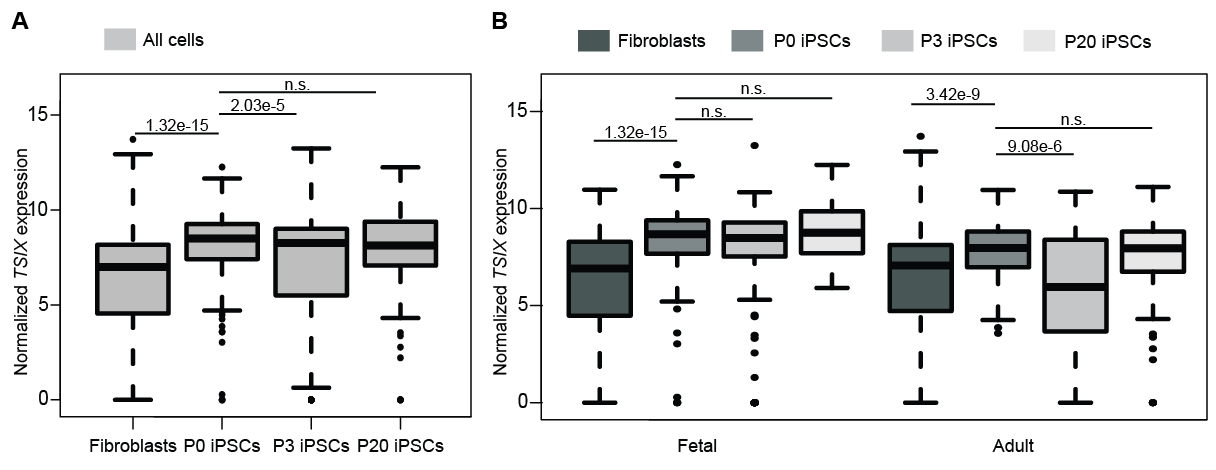


**Figure S8. *TSIX* expression**

**(A)** TSIX expression in fibroblasts through P20 iPSCs. Fibroblasts have lower levels than P0 iPSCs. While expression decreases relative to P0 at P3, expression returns to P0 levels by P20.

**(B)** TSIX levels broken down by cell line. Fetal iPSCs did not differ in TSIX expression over 20 passages. Adult iPSCs drive the difference seen in A, as levels decrease at P3.

**Supplemental Table 1: Evagreen Primer Sequences Used in This Study, Related to Figure 1,2,3, S2, S8**

| **Name** | **Forward Sequence** | **Length** | **Reverse Sequence** | **Length** |
| --- | --- | --- | --- | --- |
| *CTNNB1* | AGCTCTTACACCCACCATCC | 20 | TGCATGATTTGCGGGACAAA | 20 |
| *XIST* | AGCTCCTCGGACAGCTGTAA | 20 | GGACACATGCAGCGTGGTA | 19 |
| *SRY* | AGCTGGGATACCAGTGGAAAA | 21 | TCTCTGTGCATGGCCTGTAA | 20 |
| *RBMY1D* | TCTGCTGTGGCAAGAAGCAA | 20 | TCTGCGTGGAGGAACTCCATA | 21 |
| *CD99* | CACCGAACCCACCCAAAC | 18 | CGCAAGGTCAGCATCTGAAA | 20 |
| *ATRX* | TGAGAAGTGGCAAGAGGGATTA | 22 | TCTCCTGAGGACGTTTCACA | 20 |
| *HPRT1* | GCTTTCCTTGGTCAGGCAGTA | 21 | ACTTCGTGGGGTCCTTTTCAC | 21 |
| *TSIX* | AGCCTGGGAAACATGGTGAA | 20 | GACTACAGGTCCACACAACCA | 21 |
